# A MiSeq-HyDRA platform for enhanced HIV drug resistance genotyping and surveillance

**DOI:** 10.1038/s41598-019-45328-3

**Published:** 2019-06-20

**Authors:** Tracy Taylor, Emma R. Lee, Mikaela Nykoluk, Eric Enns, Binhua Liang, Rupert Capina, Marie-Krystel Gauthier, Gary Van Domselaar, Paul Sandstrom, James Brooks, Hezhao Ji

**Affiliations:** 1 National HIV and Retrovirology Laboratories, National Microbiology Laboratory at JC Wilt Infectious Diseases Research Centre, Public Health Agency of Canada, Winnipeg, Canada; 2 Bioinformatics Core, National Microbiology Laboratory, Public Health Agency of Canada, Winnipeg, Canada; 3 Department of Biochemistry and Medical Genetics, University of Manitoba, Winnipeg, Canada; 4 Department of Medical Microbiology and Infectious Diseases, University of Manitoba, Winnipeg, Canada

**Keywords:** Genetics, Next-generation sequencing

## Abstract

Conventional HIV drug resistance (HIVDR) genotyping utilizes Sanger sequencing (SS) methods, which are limited by low data throughput and the inability of detecting low abundant drug resistant variants (LADRVs). Here we present a next generation sequencing (NGS)-based HIVDR typing platform that leverages the advantages of Illumina MiSeq and HyDRA Web. The platform consists of a fully validated sample processing protocol and HyDRA web, an open web portal that allows automated customizable NGS-based HIVDR data processing. This platform was characterized and validated using a panel of HIV-spiked plasma representing all major HIV-1 subtypes, pedigreed plasmids, HIVDR proficiency specimens and clinical specimens. All examined major HIV-1 subtypes were consistently amplified at viral loads of ≥1,000 copies/ml. The gross error rate of this platform was determined at 0.21%, and minor variations were reliably detected down to 0.50% in plasmid mixtures. All HIVDR mutations identifiable by SS were detected by the MiSeq-HyDRA protocol, while LADRVs at frequencies of 1~15% were detected by MiSeq-HyDRA only. As compared to SS approaches, the MiSeq-HyDRA platform has several notable advantages including reduced cost and labour, and increased sensitivity for LADRVs, making it suitable for routine HIVDR monitoring for both patient care and surveillance purposes.

## Introduction

Successful antiretroviral therapy (ART) serves to suppress HIV viral load (VL), reduce the incidence of new infections, and increase the life expectancy of infected individuals^1,2^. HIV-infected patients adhering to a prescribed ART regimen now have close to normal life expectancies and maintain viral suppression at levels that mitigate transmission of the virus^3^. However, HIV drug resistance (HIVDR) can emerge as a result of poor proof-reading during HIV replication and/or unsuccessful ART due to ineffective ART regimens, poor adherence to treatment, or prolonged usage of a failing regimen^4^. The presence of drug resistance (DR) variants, including low-abundance drug-resistant variants (LADRVs), can significantly reduce ART efficacy and present a risk for treatment failure^5–10^. At the population level, transmitted HIVDR variants can have a considerable impact on the effectiveness of currently available antiretroviral drugs. Effective HIVDR monitoring and surveillance programs are essential for informing clinical care of individual patients and for strategic ART recommendations at population levels^4,11^.

Conventional HIVDR genotyping employs Sanger sequencing (SS) technology, which reliably detects DR mutations present at frequencies of ≥20% in HIV quasispecies^6,10^. However, SS is limited by its low throughput, failure to resolve codons with multiple mixed bases, and limited ability to accurately detect variants present at frequencies below 20%^12^. Recently, there has been an increased focus on the clinical significance of LADRVs on the effectiveness of ART^10,13^. It has been shown previously that patients with treatment failure had been infected with viruses that harboured pre-existing drug resistance variants ranging from 0.07 to 2.0%, as measured by allele-specific real-time polymerase chain reaction^14^. Furthermore, Pennings estimated that the probability of drug resistance evolving due to pre-existing genetic variation to be as high as 39%, depending on the antiviral treatment^15^. Recent studies demonstrated that lowering drug-resistance mutations (DRM) detection threshold from 20% to 5% improved the identification of patients at risk of virological failure^16,17^ and, depending on a drug regimen, was significantly associated with a longer time to viral suppression^16^. Therefore, more sensitive methods for routine detection of LADRVs are required in order to accurately inform individual treatment and population surveillance of transmitted HIVDR.

Since their introduction in 2005, next generation sequencing (NGS) technologies have evolved considerably, driven by the demand for low cost and high throughput genome sequencing, resulting in the commercialization of multiple NGS platforms. Although their technical designs and sequencing chemistries vary, all NGS technologies share a paradigm of parallelism, clonal sequencing and high data output. These features enable all NGS platforms to sequence templates with higher throughput, improved sensitivity for minor variants and significantly lowered per sample cost in large batched sample runs making them preferred methods for HIVDR genotyping^18^.

Roche 454 pyrosequencing technology was the first NGS technology commercialized in 2005. With the longest read lengths, 454 pyrosequencing dominated the NGS field for many years. The major disadvantage of the 454 pyrosequencing platform had been its difficulty to accurately sequence through stretches of homopolymer regions^19,20^. A similar limitation also exists to Ion Torrent semiconductor sequencing, another NGS technology that relies on the proportional increase of signal strength for enumerating long nucleotide repeats^18^. Notably, seventeen HIV DRMs are located in or near homopolymer regions in the protease (PR) and reverse transcriptase (RT) genes^19^. A platform that can accurately detect variations at these sites is required due to their importance in defining HIVDR profiles.

More recently, improved NGS technologies have been developed that provide similar read length, increased coverage, higher resolution for homopolymer regions, and increased sensitivity for detection of low-frequency variants^21^. One such platform is the Illumina MiSeq, which employs reversible-terminator chemistry and sequencing-by-synthesis technology and has good accuracy for sequencing homopolymeric regions.

Ultra-deep sequencing methodologies produce large data sets and analysis of such data requires extensive computational resources. Processing and interpreting NGS-derived data also require extensive knowledge of bioinformatics and scripting analysis pipelines. Several proprietary pipelines have been developed to address the needs for automated NGS-based HIV DR genotyping^22,23^. Our group developed an HIVDR analysis tool called HyDRA Web (http://hydra.canada.ca), which is highly comparable to other HIVDR pipelines and has been freely available for all internet users since March 2016^24^. HyDRA Web generates a report on all HIVDR mutations found in the *pol* genes (PR, RT, IN) by referring to Stanford HIVDR database algorithms^25–27^, as well as a consensus sequence at a user-defined threshold, complete nucleotide and amino acid variant frequency reports, and read pileups in standard binary alignment map (BAM) file format for further downstream analysis^24^.

**Figure 1:**
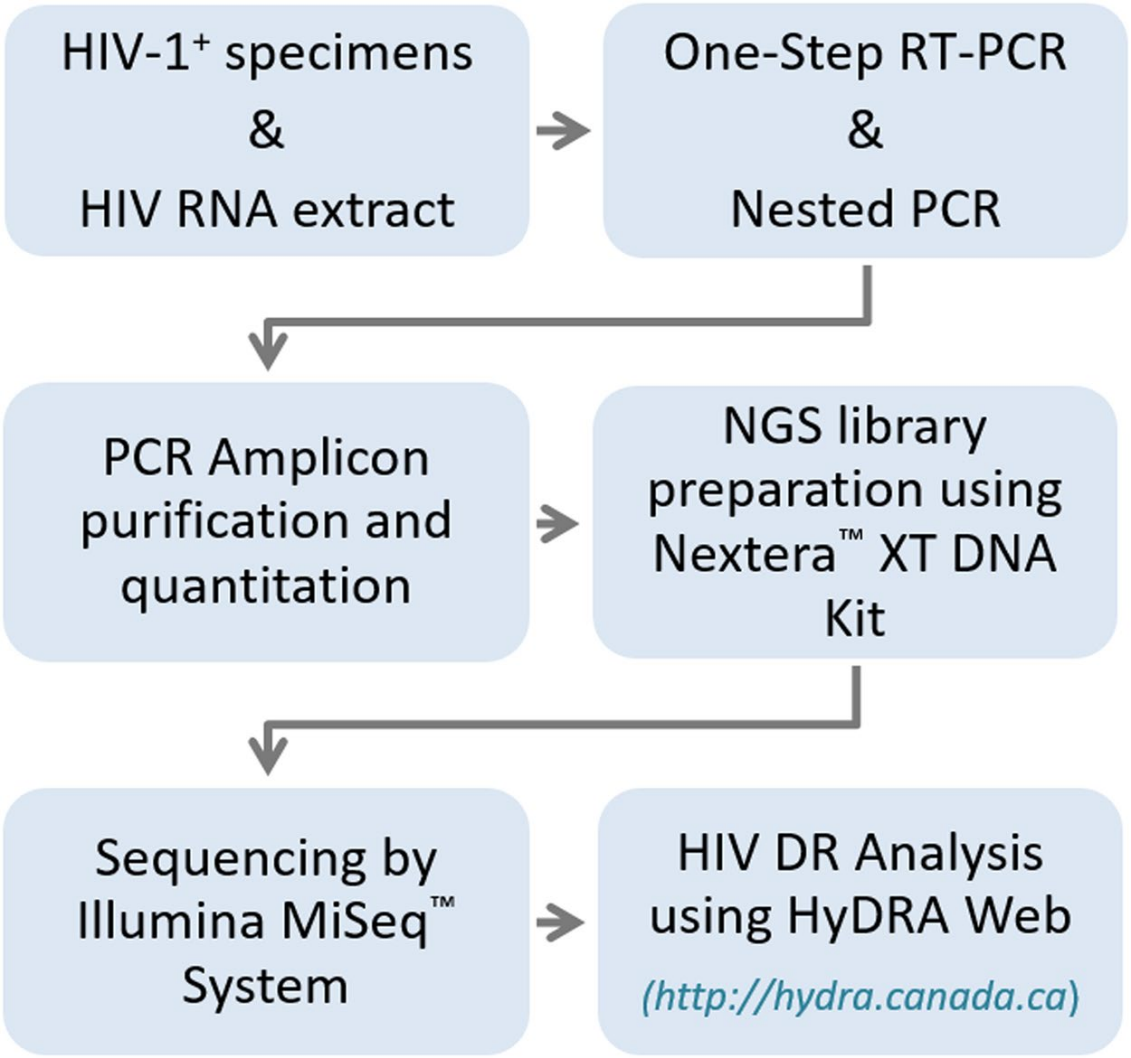
Miseq-HyDRA platform workflow.

Following the recommendations for validating an HIV genotyping assay as described by the WHO^28^ and a newly proposed NGS HIVDR assay assessment system^29^, we characterized the performance of a new MiSeq-HyDRA platform for its accuracy, precision, reproducibility, and sensitivity in identifying HIVDR mutations in the PR and RT regions within major HIV-1 subtypes. Here, we describe our validated MiSeq-HyDRA platform (Fig. 1) which includes detailed sample processing and NGS-based HIVDR genotyping protocol for all major HIV-1 subtypes. Our validation panel consisted of HIV-spiked plasma, two commercially prepared plasmids, and clinical and proficiency specimens. Specimens at VLs of ≥1,000 copies/ml (cp/ml) were consistently amplified for all examined subtypes. The MiSeq-HyDRA protocol identified all HIVDR mutations found by SS, however in contrast to SS, only the MiSeq-HyDRA platform identified LADRVs at frequencies between 1~15%. Plasmid mixtures were used to determine the gross error rate and the detection limit for LADRVs in and outside of homopolymer regions. The MiSeq-HyDRA platform is a favoured alternative to SS for HIVDR genotyping in clinical and surveillance settings because of its increased sensitivity, cost and labour reduction, and superior detection of LADRVs in homopolymer regions.

## Methods

### Samples

A panel of HIV-spiked plasma with known VLs was obtained from the External Quality Assurance Program Oversight Laboratory (EQAPOL, Duke University, USA) and used to test the subtype specificity and sensitivity of our primers. The EQAPOL panel included 2 samples from each of the following subtypes; A1, B, C, D, G, F, CRF01_AE, CRF02_AG. Certificates of Analysis for each EQAPOL sample provided the reference viral load (Roche COBAS methodology). Two commercially prepared plasmids with known HIVDR mutations, P1 (G73T_K103N) and P2 (G73T_K65R) (Genscript, Piscataway, USA), were used to assess the error rate of the assay, as well as the limit of detection for minority variants in a range of P1:P2 mixtures.

Further characterization and validation were performed using a small cohort of anonymized clinical specimens (n = 58) obtained through the Research Ethics Board Exempt Strain and Drug Resistance (SDR) Surveillance Program. These clinical specimens were collected in 2012 and 2013 from treatment–naïve HIV-1 positive patients with unknown VL, and selected for testing on our MiSeq-HyDRA platform to represent a variety of clades and HIVDR mutations. Also included were two panels, each consisting of 5 HIV-1 positive plasma samples with known VL (n = 10), from the Virology Quality Assurance (VQA) Program (Rush University Medical Center, USA), originally acquired for HIVDR genotyping proficiency test. All clinical and panel samples were previously sequenced using an in-house VQA-validated SS protocol and represented all major HIV-1 subtypes including; A, B, C, D, F, G, CRF01_AE, CRF02_AG, CRF06_cpx, CRF12_BF, as well as two A1/D and one G/B recombinant viruses.

### HIV RNA extraction

For all samples tested, total nucleic acid was extracted from 400 μl of HIV-1 infected plasma and eluted in 110 ul using the Nuclisens EasyMag system (Biomerieux, St-Laurent, Canada) according to the manufacturer’s suggested protocol. The EQAPOL panel of HIV-spiked plasma was serially diluted using normal human plasma (NHP), prior to HIV RNA extraction, to represent a range of VL from 10,000 cp/ml to 50 cp/ml. An extraction efficiency of 90% was estimated from previous data (not shown here) in order to calculate the approximate viral RNA copy number in each RT-PCR reaction. For the clinical SDR specimens and VQA samples, the same RNA extract used for SS was used for preparing amplicons towards sequencing on the MiSeq.

### Sample amplification

RT-PCR was performed on 10 µl of each extract of the EQAPOL, VQA, and clinical samples using Superscript^™^ III One-Step RT-PCR Platinum^®^ Hi-Fidelity Taq System (Thermo Fisher Scientific, Canada), according to the manufacturer’s suggested protocol. The primers used were as follows: PR_F1 5′-GARAGACAGGCTAATTTTTTAGGGA-3′ (HXB2 loci 2071–2095) and RT_R1 5′-ATCCCTGCATAAATCTGACTTGC-3′ (HXB2 loci 3348–3370). RT-PCR conditions were performed as follows: 50 °C for 30 minutes, 94 °C for 2 minutes, 40 cycles of 94 °C for 20 seconds, 50 °C for 30 seconds and 68 °C for 90 seconds, and a final extension at 68 °C for 5 minutes. The same RT-PCR conditions were also used to prepare amplicons for Sanger sequencing^30^.

Following RT-PCR, a 5 µl aliquot was transferred to a nested-PCR reaction, performed using Phusion^®^ Hot Start II Hi-Fidelity DNA Polymerase (Thermo Fisher Scientific, Canada), according to the manufacturer’s recommended protocol. The primers used were as follows: PR_F2 5′-CTTTARCTTCCCTCARATCACTCT-3′ (HXB2 loci 2243–2266) and RT_R2 5′-CTTCTGTATGTCATTGACAGTCC-3′ (HXB2 loci 3304–3326). Nested-PCR conditions were performed as follows: 98 °C for 30 seconds, 35 cycles at 98 °C for 10 seconds, 62 °C for 20 seconds and 72 °C for 40 seconds, followed by a final extension at 72 °C for 10 minutes^30^.

Plasmids were amplified by one round of PCR according to the above conditions for nested-PCR. All PCR products were qualitatively confirmed using QIAxcel^®^ Capillary Electrophoresis (Qiagen, Canada).

### Sanger sequencing and analysis

Briefly, the derived amplicons were purified using MultiScreen^®^ HTS PCR 96 filter plate (Millipore, Canada), qualitatively assessed and quantified by QIAxcel^®^ (Qiagen, Canada), and subject to sequencing PCR using the ABI Prism BigDye^®^ cycle sequencing system and ABI 3730 DNA Analyzer (Thermo Fisher Scientific, Canada). Eight sequencing primers provided bi-directional coverage of the entire PR/RT amplicon. The derived sequences were then assembled using RECall^31^ and alignment with the HXB2 reference sequence (Accession number: K03455). The Stanford HIV Genotypic Resistance Interpretation Algorithm (http://hivdb.stanford.edu) was used to identify subtype and mutations associated with drug resistance. Any discrepancies in subtype were resolved using the REGA v3 HIV Subtyping Tool (http://regatools.med.kuleuven.be/typing/v3/hiv/typingtool/)^32^.

### MiSeq library prep and sequencing

Amplicon concentrations were first determined for all samples using the dsDNA HS assay kit and Qubit^®^ fluorometer (Thermo Fisher Scientific, Canada) and then brought to a final concentration of 0.2 ng/µl. Purification of amplicons prior to starting the library prep was not required. All PCR reactions yielded a single specific band with no significant primer dimers. Previous tests showed that PCR purification did not improve the quality of the libraries or the final results (data not shown). Libraries were prepared using the 96-sample Nextera^®^ XT DNA Library Preparation Kit (Illumina, USA). Tagmentation, indexing, purification and library normalization were all performed according to the manufacturer’s detailed Reference Guide^33^. It was not necessary to confirm library size by the use of a Bioanalyzer. The bead-based normalization as described in the Reference Guide was optimally suited to our purpose. Libraries were pooled in equal volumes and diluted 30× in the provided hybridization buffer. A 20 pM PhiX control library (v3, Illumina, USA) was spiked in at 20%, in order to provide quality control measures and to increase the diversity of the amplicon libraries. Samples were sequenced on the MiSeq using either the v2 500-cycle or v3 600-cycle MiSeq reagent kits (Illumina, USA).

### MiSeq data analysis

MiSeq data analysis was performed using HyDRA Web (http://hydra.canada.ca), a freely available online automated pipeline for HIVDR analysis of NGS-derived data^24^. The HyDRA pipeline is coded in Python and leverages existing open source bioinformatics software for annotated reference-based mapping and variant calling against the HXB2 *pol* gene (loci 2253–5096, GenBank Accession number: K03455). The pipeline carries out the multi-step data processing starting from raw NGS data input (in.fastq,.fastq.gz, or.sff formats) to customizable reporting on HIVDR mutations at any defined frequency. Advanced options for data quality assurance, filtering, variant calling, and reporting thresholds are modifiable to customize the analysis.

For our analysis, we used the HyDRA Web default settings for analysis, unless otherwise indicated^24^. Briefly, reads were first filtered by a minimum quality score of Q30 and 100 bp length, mapped using Bowtie2 with the reference sequence (HXB-2 or known plasmid sequences). Variants were called based on a pre-determined error rate of 0.0021 for the MiSeq platform (see Results), a minimum variant quality of Q30, a minimum read depth of 100×, and a minimum allele count of five. HIVDR mutations detected above a 1% frequency were reported based on the default HyDRA Web Mutation Database, which is a combination of the Stanford 2015 list of HIV-1 drug resistance mutations (http://hivdb.stanford.edu), with added annotations from the WHO 2009 list of mutations for surveillance of transmitted HIV drug resistance. HIVDR mutations are reported for reverse-transcriptase and protease using the Stanford classification designations: Major, Accessory or Other ((http://hivdb.stanford.edu). Consensus sequences were generated with thresholds of 20%, 15%, and 10% for genotypic and phylogenetic comparison to corresponding Sanger data.

### Statistical analysis

MiSeq-derived data for the pedigreed plasmids was analyzed for variant frequencies using a local install Galaxy with tools to apply Q30 quality filters to the FASTQ files, Bowtie2 for reference mapping, and an in-house tool for collating variant frequencies to a CSV file for further statistical analysis in Microsoft Excel. Clinical samples sequenced by both Sanger and MiSeq-based assays were analyzed for nucleotide and amino acid percent identity using MEGA6.0^34^.

## Results

### HIV-1 subtype coverage and viral load limit of detection

The HIV subtype coverage of any given protocol depends on the initial HIV viral RNA extraction and PCR amplification steps that render templates for subsequent HIV genotyping. The subtype specificity and sensitivity of the new protocol presented here were first assessed using an EQAPOL panel of HIV-spiked plasma with known viral load, including eight major HIV-1 subtypes, A1, B, C, D, F, G, CRF01_AE, and CRF02_AG. A plasma dilution series, representing VLs from 10,000 cp/ml down to 50 cp/ml was processed in three independent assays over a 6-month period performed by 3 different technicians using different reagent lots. The estimated viral RNA copy number per PCR reaction, expected from each plasma dilution, was calculated based on described volumes with an assumed extraction efficiency of 90%.

As shown in Table 1, all examined major subtypes were successfully amplified in the three independent assays to a minimum of 500 cp/ml, except for subtype D, which failed to amplify at ≤1,000 cp/ml in one dilution series. In addition, a subset of clinical samples of unknown viral load previously tested for HIVDR were also examined in this study and all these specimens were successfully amplified and eventually sequenced. HIV-1 subtypes in the clinical subset included subtype A, B, C, D, E, F, G, CRF01_AE, CRF02_AG, CRF50_A1D and, CRF06_cpx.

**Table 1:** Successful (+) or unsuccessful (−) PCR amplifications of 8 major HIV-1 subtypes in three independent assays.

HIV-1 Subtype	Plasma VL (RNA copies/ml)	10,000	5,000	2,500	1,000	500	200	100	50
	*RNA copy # per RT-PCR reaction	330	165	82.5	33	16.5	6.6	3.3	1.6
A1		+++	+++	+++	+++	+++	−++	+++	−++
CRF01_AE		+++	+++	+++	+++	+++	+++	+++	−−+
CRF02_AG		+++	+++	+++	+++	+++	+−−	−−−	−−+
B		+++	+++	+++	+++	+++	+++	−−+	−−−
C		+++	+++	+++	+++	+++	−−+	+++	−−−
D		+++	+++	+++	++−	++−	−−−	−−−	−−−
F2		+++	+++	+++	+++	+++	+++	−+−	−−−
G		+++	+++	+++	+++	+++	+++	++−	++−

*Estimated viral RNA copy number in the PCR was calculated based on 90% extraction efficiency.

### Error rate as determined using pedigreed plasmids

To accurately assess the gross error rate associated with the MiSeq-HyDRA platform, including PCR amplification, sequencing and data processing steps, we used two commercially prepared plasmids constructed from the PR-RT region of the HXB2 *pol* gene, each with two DRMs: P1 (G73T_K103N) and P2 (G73T_K65R) (Genscript, USA). Both plasmids were processed using the same nested-PCR, MiSeq library preparation and sequencing protocol in triplicate intra-assays and triplicate inter-assays (see Methods). Using an in-house developed Galaxy pipeline, MiSeq-derived FASTQ files were filtered using the same parameters applied by the HyDRA pipeline; i.e. a minimum Q30 quality and read length of 100, and mapped using Bowtie2 with the known plasmid reference sequence^35–37^. All detected discrepancies from the plasmid reference sequences were recorded as errors derived from the workflow.

To calculate the error rate for each plasmid, we first determined the error rate at each nucleotide position (n = 974) by dividing the sum of mismatched variant base calls, insertions, and deletions by the total number of base calls at that locus. The variant frequencies calculated for each nucleotide position were then averaged to obtain an error rate for each plasmid (Supplementary Table S1). The overall gross error rate for the MiSeq-HyDRA platform was then calculated by averaging the error rate obtained from each plasmid and was determined to be 0.21%, with a standard deviation (SD) of 0.01%.

To further assess the suitability of MiSeq-HyDRA platform for HIVDR genotyping, we calculated error rates at HIV DRM codons and at homopolymeric genomic regions. The forty-three PR and RT codons identified as surveillance drug-resistance mutation (SDRM) sites by the Stanford HIVDR database (https://hivdb.stanford.edu/) were taken into account in this analysis. Homopolymeric loci were defined as a succession of three or more identical nucleotides. For the SDRM loci, the mean error rate was determined to be 0.21% (SD = 0.01%), essentially the same as observed for the full plasmid amplicon sequence. The mean error rate at the homopolymeric loci was determined to be 0.24% (SD = 0.01%). Comparable results were obtained when these plasmids were tested in three independent assays conducted by three different technicians, showing excellent reproducibility (Supplementary Table S1).

### Sensitivity for minor HIV-1 DRM detection in artificial plasmid mixtures

Artificial mixtures of the aforementioned P1 and P2 plasmids harbouring distinct DRMs were prepared and used to assess the sensitivity of the MiSeq-HyDRA platform for detecting minor variants in a mixed population. Pure plasmids, P1 (G73T_K103N) and P2 (G73T_K65R), were mixed at various ratios and the resulting plasmid mixtures were used for 3 independent PCR amplifications and library preparations, and then sequenced on a single MiSeq run.

As expected, the G73T variant, which is present in both plasmids, is detected at >99% in the pure plasmids as well as in all the mixtures (Table 2). In contrast, the frequency of the K103N substitution is detected at >99% in the pure P1 plasmid, and then sequentially decreases as the ratio of P2 increases in the mixtures. The same pattern was observed for K65R derived from P2. When present as the minor variant in the mixture, K103N was consistently detected to a minimum average frequency of 0.53%, but was not detected in any of the replicates at 0.1%. Similarly, the minor variant K65R in P2 was consistently detected at an average minimum frequency of 0.61%, but due to imprecision in the 99.9:0.1 plasmid mixture (possibly due to a combination of mixture quantitation error and sequencing error) the 0.1% frequency was not accurately assessed. More importantly, analysis of all the mixed plasmid sequence data by HyDRA Web revealed no other amino acid substitutions above 0.5% apart from the known expected variants. Based on the observed error rate of 0.21% and the ability of the assay to reliably detect minor variants at >0.5%, the default setting for minor variant reporting by HyDRA Web was conservatively set to 1%.

**Table 2:** Sensitivity for the detection of minor variants in mixed plasmid populations by the MiSeq-HyDRA platform. *n/d = not detected.

Plasmid Mixture (%)		P1	M1	M2	M3	M4	M5	M6	M7	M8	M9	M10	M11	M12	M13	P2
P1 (G73T_K103N)		100	99.9	99.5	99	95	90	80	50	20	10	5	1	0.5	0.1	0
P2 (G73T_K65R)		0	0.1	0.5	1	5	10	20	50	80	90	95	99	99.5	99.9	100
**Mutation**	**Replicate**	**Variant Frequency Detected (%)**														
G73T(P1 & P2)	1	99.62	99.59	99.61	99.65	99.64	99.65	99.68	99.57	99.62	99.60	99.61	99.59	99.61	99.56	99.50
	2	99.58	99.60	99.64	99.49	99.65	99.62	99.55	99.17	99.56	99.55	99.58	99.55	99.57	99.65	99.61
	3	99.61	99.56	99.60	99.55	99.60	99.55	99.54	99.57	99.60	99.60	99.62	99.53	99.56	99.52	99.65
	**AVG**	**99.60**	**99.58**	**99.62**	**99.56**	**99.63**	**99.61**	**99.59**	**99.44**	**99.59**	**99.58**	**99.60**	**99.56**	**99.58**	**99.58**	**99.59**
K103N (P1)	1	99.11	98.90	98.36	97.69	92.06	85.42	73.81	48.51	21.66	11.66	5.88	1.25	0.50	n/d	n/d
	2	99.34	98.92	98.59	97.71	92.29	85.91	72.95	48.06	21.42	11.50	6.03	1.15	0.60	n/d	n/d
	3	99.12	98.74	98.21	97.52	91.45	84.54	72.30	48.59	21.54	12.39	5.50	1.17	0.49	n/d	n/d
	**AVG**	**99.19**	**98.85**	**98.39**	**97.64**	**91.93**	**85.29**	**73.02**	**48.39**	**21.54**	**11.85**	**5.80**	**1.19**	**0.53**	n/d	n/d
K65R (P2)	1	n/d	0.58	1.23	2.02	7.50	14.82	25.73	49.97	74.60	84.75	92.91	98.05	98.64	99.15	99.26
	2	n/d	0.62	1.16	1.77	7.49	14.29	26.75	48.44	76.34	86.50	92.70	97.76	98.75	99.28	99.24
	3	n/d	0.64	1.13	2.04	8.58	15.10	26.79	48.85	75.90	85.43	92.72	97.95	98.79	99.15	99.74
	AVG	n/d	**0.61**	**1.17**	**1.94**	**7.86**	**14.74**	**26.42**	**49.09**	**75.61**	**85.56**	**92.78**	**97.92**	**98.73**	**99.19**	**99.41**

### Performance characteristics of the MiSeq-HyDRA platform and concordance with Sanger consensus sequences on clinical specimens

To further assess the performance characteristics and clinical suitability of this new platform for HIVDR genotyping, we analyzed 68 clinical samples, consisting of a cohort of 58 SDR specimens and two 5-member VQA genotyping panels, using SS and the MiSeq-HyDRA protocol.

#### Consensus sequence concordance

To assess the ability of the MiSeq-HyDRA platform to simulate SS reads, we compared the concordance of the NGS consensus sequences generated from HyDRA with the corresponding SS sequences from a cohort of clinical specimens. HyDRA Web allows for consensus sequences to be generated from the MiSeq sequencing data at a user-defined mixed base calling threshold. The percent nucleotide identity between SS and MiSeq-HyDRA consensus sequences was determined using three different thresholds for the MiSeq sequence data: 20%, 15% and 10%. Overall, the concordance is high between the sequencing platforms. The percent nucleotide identity is >99% at all three thresholds, and decreases only marginally as the threshold decreases, from 99.62% with 20% threshold, to 99.58% with 15% threshold, to 99.35% with 10% threshold (Supplementary Table S2). The majority of nucleotide discordance between MiSeq-HyDRA consensus and matching SS sequence was due to either a mixed base call or a component of that mixed base, with only a fraction from complete base changes.

#### Accuracy

Accuracy in HIVDR genotyping refers to the ability of the assay to detect known HIV DRMs by using conventional SS readouts as a standard benchmark. Using the same RT-PCR amplicon that was used for SS, we compared the HyDRA Mutation Reports for each clinical sample obtained from the MiSeq-HyDRA assay with that of the Stanford Genotypic Resistance Report derived from the same samples using our in-house SS protocol. The HyDRA report accurately identified 100% of the 166 HIV-1 DRMs previously detected by SS, 67 of which were classified as surveillance drug resistance mutations (SDRMs) (Supplementary Table S3). In addition, the MiSeq-HyDRA assay identified another 84 HIV DRMs in this sample cohort, 33 of which classified as SDRMs, at frequencies ranging from 37% down to 1%, which were not identified in the SS data (Supplementary Table S3). The majority of these mutations (n = 76) were detected at frequencies <10%. However, three of these discrepancies between the MiSeq-HyDRA data and SS occurred at frequencies >20% (Table 3), challenging the generally accepted detection limit for SS of 20%^6,10^.

**Table 3:** Comparison between the MiSeq-HyDRA platform and SS for detection of HIVDR-related mutations near the conventional 20% threshold. SDRMs are shown in **bold**.

Sample ID	Clade	Gene	DRM	Classification	MiSeq Frequency	Detected by SS	Viral Load (cp/ml)	Estimated PCR copy #
VQA29-5	B	PR	L10I	Other	37.15	NO	8,182	270
VQA29-4	B	PR	L10F	Accessory	34.99	YES	6,849	225
SDR-112	A1/D*	RT	**L74I**	NRTI	34.70	YES	unknown	unknown
VQA29-3	C	RT	V90I	Other	33.18	YES	15,101	500
VQA30-3	B	PR	**I54L**	Major	30.68	YES	6,508	215
SDR-112	A1/D9*	RT	E138K	NNRTI	29.41	NO	unknown	unknown
VQA29-5	B	PR	**I54L**	Major	29.21	YES	8,182	270
VQA29-4	B	PR	L10V	Other	27.66	YES	6,849	225
SDR-89	A	PR	K20R	Other	26.11	NO	unknown	unknown
SDR-102	B	RT	**G190A**	NNRTI	21.02	YES	unknown	unknown
SDR-121	A1	RT	V106I	Other	19.85	YES	unknown	unknown
SDR-36	B	RT	**K219Q**	NRTI	18.93	YES	unknown	unknown
SDR-95	CRF01_AG	PR	L33F	Accessory	17.54	YES	unknown	unknown
SDR-109	CRF01_AE	PR	L10I	Other	17.30	NO	unknown	unknown
SDR-112	A1/D*	RT	T69N	Other	17.22	YES	unknown	unknown
VQA30-3	B	PR	L10I	Other	15.91	YES	6,508	215
SDR-119	B	PR	**M46I**	Major	15.57	NO	unknown	unknown
SDR-34	F	RT	V106I	Other	15.08	NO	unknown	unknown
SDR-102	B	RT	V179I	Other	13.30	NO	unknown	unknown
SDR-95	CRF01_AG	RT	K101R	Other	10.68	YES	unknown	unknown
SDR-117	B	RT	**L210W**	NRTI	9.21	YES	unknown	unknown
VQA29-3	C	RT	**M41L**	NRTI	8.27	NO	15,101	500

Another 75 mutations at frequencies 1~8.13% that were not detected by SS were shown in Supplementary Table S3. ^*^Recombinant assignments are based on PR/RT sequence data.

Specifically, sample VQA29- 5 (Table 3), taken from the VQA HIV DR Genotyping proficiency panel, was tested by 43 independent laboratories using either an in-house SS-based genotyping protocol or the Viroseq Genotyping System (Abbott). Only 3 out of 43 labs reported the DRM L10I, according to the VQA panel report. Similarly, the clinical sample SDR-89 was sequenced in duplicate by our SS-based method and the DRM K20R was never detected by the RECall analysis software. In contrast, SDR-89 and DRM K20R were also used to validate the inter-assay reproducibility of the MiSeq-HyDRA platform as shown in Table S5. For SDR-112, there was only sufficient material for a single run on each platform, SS and MiSeq. In addition, several other mutations between 9~20% were detected by SS (Table 4), indicating that a 20% threshold consensus sequence is not ideal to use for DRM analysis.

**Table 4:** Intra-assay precision for LADRVs identified by the MiSeq-HyDRA assay.

Sample	Gene	Class	DRM	Rep1^¶^	Rep 2^¶^	Rep3^¶^	SS*
SDR-55	PR	Other	K20I	98.97	98.78	99.06	YES
	PR	Other	V82I	99.25	99.24	99.21	YES
	RT	Other	V90I	**2.83**	ND	ND	NO
	RT	Other	V118I	ND	**9.88**	ND	NO
	RT	Other	V179I	**1.44**	**7.98**	**1.38**	NO
	RT	**NRTI**	M184V	99.34	99.08	99.19	YES
	RT	Other	L210S	2.44	ND	ND	NO
SDR-95	PR	Other	V11I	94.58	96.59	95.85	YES
	PR	Other	K20I	99.31	97.07	99.18	YES
	PR	Accessory	K20T	ND	**2.2**	ND	NO
	PR	Accessory	L33F	**17.54**	**29.3**	**17.88**	**YES**
	PR	**Major**	M46I	ND	**1.47**	ND	NO
	PR	Other	V82I	42.21	44.48	35.9	**YES**
	RT	NRTI	K65E	ND	ND	**1.86**	NO
	RT	Other	K101R	**10.68**	**9.04**	**8.38**	YES
	RT	Other	V106I	**1.55**	ND	ND	NO
	RT	NNRTI	V108I	ND	**1.04**	ND	NO
SDR-140	PR	Other	L10I	98.69	98.63	98.74	YES
	PR	**Accessory**	N88D	ND	**6.89**	ND	NO
	RT	**NRTI**	L74V	ND	**1.13**	ND	NO
	RT	**NNRTI**	K103N	99.41	99.08	99.41	YES
	RT	**NNRTI**	P225H	98.95	83.73	99.12	YES
SDR-143	RT	NRTI	A62V	ND	**3.11**	ND	NO
	RT	**NNRTI**	K103N	99.3	99.39	99.35	YES

SDRMs are highlighted in **bold**. ^¶^Mutation frequencies (%) detected in replicates; *Detection by Sanger-based sequencing assay.

#### Precision and reproducibility

Precision measures the ability of an assay to generate the same result on replicates of the same sample within a test run (intra-assay variability). In contrast, reproducibility measures the ability of an assay to generate the same result on the same sample in different test runs (inter-assay variability)^28,29^. Both parameters are used to measure the performance of a given assay. To assess the precision and reproducibility of MiSeq-HyDRA on clinical specimens, a subgroup of well-characterized specimens (n = 7) were analyzed in triplicate intra- and inter-assays (see Methods). The concordance between MiSeq-HyDRA consensus sequences at 3 different thresholds: 20%, 15%_,_ and 10%_;_ and the matching SS sequences were recorded. We observed high concordance (≥99.40%) for both nucleotide and amino acid consensus sequences between the two platforms at all 3 thresholds for both intra- and inter-assay performance (Supplementary Table S4). When comparing consensus sequences with a 20% threshold from MiSeq-HyDRA and their corresponding Sanger sequences, the few discordant nucleotides and amino acids among all examined specimens were all due to mixed base calls. Notably, four of these discrepancies were found in HIVDR-associated codons resulting in two silent substitutions (data not shown), and two DRMs, both of which were identified in specimen SDR-95. The HyDRA report revealed the frequencies of the 2 discrepant HIVDR-related substitutions, L33F in PR and K101R in RT, in SDR-95 replicates (Table 4). Though neither amino acid substitution affected the overall HIVDR profile for that individual, it demonstrates the need for a lower threshold when defining a consensus sequence that encompasses all potentially relevant HIVDR mutations.

The precision and reproducibility of the MiSeq-HyDRA assay were further assessed by examining its consistency in detecting the frequency of DRMs. Table 4 illustrates in detail the frequencies associated with LADRVs detected in 4 out of the 7 clinical specimens used in the intra-assay comparison. The remaining 3 samples harboured HIV DRMs at >99% frequencies, with 100% precision in intra-assay triplicates (data not shown). In contrast, a lack of precision for detecting LADRVs <10% is observed in specimens SDR-55, 95, 140, and 143 (Table 4). The results from inter-assay comparisons of another 7 clinical specimens (Supplementary Table S5), further supports this observation that reproducibility is 100% for mutations present at or greater than 10% frequency. These results indicate that reliable detection of LADRVs begins at frequencies >10%, even with coverage at all sites of 20,000× (data not shown). Given the relatively low average error rate of our MiSeq-HyDRA platform of 0.2%, we do not consider these LADRVs to be false-positives, but rather to be false-negatives when not detected within all replicates. These findings highlight the importance of viral RNA copy number extracted from samples with unknown VL and the effect of PCR bias on variant frequencies. This interpretation is further supported by data obtained from inter-assay experiments (Supplementary Table S5).

#### Effect of viral load on detection of LADRVs

To evaluate the minimum VL required to detect LADRVs we analyzed the sequences of HIV-spiked plasma specimens from EQAPOL over a range of serially diluted plasma, representing viral loads of 5000, 500, and 100 cp/ml. Table 5 describes five of the samples that harboured LADRVs, defined as a frequency below 10%. Adopting a conservative extraction efficiency of 90%, we calculated the approximate viral RNA copy number per PCR reaction expected from each diluted specimen. Following our protocol, a plasma VL of 5000, 500, and 100 cp/ml approximately correspond to 160, 16, and 3 viral RNA copies/PCR reaction, respectively. Though our test group is small (n = 5), due to the fact that we had a limited number of specimens with both known VL and LADRVs, our results are as anticipated. We detected LADRVs only in the samples with higher VLs.

**Table 5:** Effect of viral load and PCR copy number on the detection of LADRVs by the MiSeq-HyDRA platform. SDRMs are in **bold**.

Plasma viral load (cp/ml)				5000	500	100
Approx. copies/PCR*				160	16	3
EQAPOL specimen	Gene	Class	DRM	Frequency (%)		
CRF01_AE	PR	**Major**	I47V	**1.17**	**ND**	**ND**
	PR	Accessory	L10V	99.5	99.71	99.84
	RT	Other	K238R	99.2	99.48	99.76
CRF02_AG	PR	Other	K20I	99.86	99.64	99.89
	RT	NNRTI	M230I	**5.12**	**ND**	**ND**
	RT	NNRTI	E138K	**ND**	**ND**	**1.08**
C	RT	**NRTI**	D67E	**2.49**	**ND**	**ND**
	PR	Accessory	K20R	99.26	99.44	99.27
F2	RT	**NNRTI**	V106A	**2.41**	**ND**	**ND**
	PR	Accessory	L10I	98.62	99.13	99.33
	PR	Accessory	K20R	99.5	99.61	99.62
G	RT	**NNRTI**	G190E	**4.08**	**ND**	**ND**
	PR	Other	K20I	99.4	99.48	99.55

*Approximate viral RNA copies per PCR reaction were calculated based on an assumed extraction efficiency of 90%.

For example, the 5% variant M230I in the CRF02_AG specimen is only detected when the initial plasma VL is 5000 cp/ml, equivalent to approximately 160 viral RNA copies/PCR reaction. At VLs of 500 cp/ml and lower, this variant would be present at <16 copy/PCR reaction, and as expected, was not detected in this dilution (Table 5). The same holds true for the other LADRVs with frequencies of 1~4%; these LADRVs are only detected in the samples with VL of 5000 cp/ml with the exception of E138K at 1.08% in the CRF02 AG specimen at 100 cp/ml. This variant is not found in the specimens with higher VLs and when analyzed in the context of PCR copy number, approximately 3 viral RNA copies for that VL, we conclude that this is a false positive result due to intrinsic assay error, and the ND (not detected) in the higher VL specimens is, in fact, a true positive. An additional consideration for evaluating the frequency of LADRVs is the extent of re-sampling during PCR, particularly in the range of 10–20%. In the absence of single genome sequencing methodologies which are not practical for routine HIVDR testing, we have no way of determining if these variants are in fact present at an even lower frequency in the viral quasispecies and to what extent these variants influence clinical outcomes. Therefore, it is important to note that both viral load and PCR re-sampling influence the frequency of HIV DRMs and should be taken into account when analyzing LADRVs.

## Discussion

HIVDR testing is recommended for persons newly diagnosed with HIV in order to guide the selection of an initial ART regimen, and also serves to provide surveillance of transmitted HIVDR in the population. In addition, testing for acquired or secondary HIVDR mutations should be performed when patients, adhering to an ART regimen, experience suboptimal virologic response or treatment failure as indicated by an increase in VL to >1000 cp/ml. Recently, there is evidence showing that low abundant drug-resistant variants (LADRVs) in the HIV quasispecies may be clinically significant and influence virologic response to ART^6,10,13–17^. Current HIVDR testing methods need to be revisited in order to satisfy the requirements of accessible, low-cost testing and an increased ability to detect LADRVs.

NGS technologies allow for ultra-deep sequencing and reliable detection of LADRVs. With one of the lowest error rates and highest read coverage of the current NGS technologies available^18,21^, the Illumina MiSeq platform is the leading candidate for routine HIVDR genotyping. This is evident in the publication of additional studies supporting the use of the MiSeq for HIVDR testing^19,38–44^.

One limitation of the use of NGS sequencing platforms is the absolute requirement and knowledge of bioinformatics tools that are essential for NGS data analysis. Many laboratories do not have the computational capacity to process the large amounts of data produced by the MiSeq or any NGS system. Bioinformatic pipelines are needed to ascertain the identity and frequency of HIVDR mutations, in particular, the LADRVs. Though no standardized method of analysis has been developed, there are several pipelines, including open source and commercial, that exist to carry out this analysis^22^. Here we introduce HyDRA Web, a free online application that gives users access to an automated and fully customizable NGS analysis pipeline for HIVDR mutations. HyDRA Web is designed to provide users with the ability to perform analysis of large NGS data sets while shielding them from the computational burden required to do so. Notably, HyDRA accommodates the NGS HIVDR analysis for HIV-1 protease, reverse transcriptase and integrase genes although only the HIV-1 integrase is not covered in this study.

Using the Illumina MiSeq platform combined with the ease of HyDRA Web data analysis, we have developed a robust, high-throughput and cost-effective HIVDR genotyping workflow that outperforms the conventional Sanger-based protocols in both sensitivity and reliability. Following the WHO guidelines for validation of HIVDR genotyping assays^28,29^, we validated our MiSeq-HyDRA platform for cross-clade specificity, viral load and minor variant sensitivity, error rate, accuracy as compared to Sanger sequencing data, and precision and reproducibility for LADRVs.

A robust PCR-based HIVDR assay should readily amplify the main M group subtypes, despite variances in genome sequences across subtypes and within a quasispecies. Dudley *et. al*. reported on cross-clade HIVDR genotyping assay for PR, RT and IN^38^. However, their protocol requires concentrating the plasma prior to extraction in order to achieve successful amplification, as well as secondary algorithms for touchdown PCR for difficult samples, additional steps which are not desirable for establishing a standardized protocol. In contrast, our assay is effective to 500 cp/ml for all examined major group M subtypes with no modifications required. Although not presented here, this platform is amendable to include IN genotyping which only requires combining PR/RT and IN amplicons prior to library prep^30^ HyDRA web already possesses the capacity to analyze IN sequence data if provided in the input Fastq files. In addition, our library prep followed the streamlined Nextera™ library prep protocol using a bead-based normalization completely satisfactory for generating coverage of at least 20,000× across the PR/RT amplicon region.

With other platforms having issues resolving sequences in homopolymer regions, it was essential to determine the MiSeq-HyDRA platform error rate with a focus in these regions. Sequence analysis of pedigreed plasmid amplicons demonstrated low error rates in the homopolymer regions which were consistent with the overall average error rate of 0.21%. In addition, MiSeq-HyDRA demonstrated low error rate in known SDRM-associated codons which were also consistent with the overall error rate of 0.21%.

LADRVs present at levels as low as 1% in quasispecies infections, can have a detrimental impact on treatment efficacy^6,10,16,17^. The MiSeq-HyDRA platform has the ability to detect variants at frequencies as low as 1%, surpassing the typical 20% threshold attributed to Sanger sequencing. Here, we have demonstrated the ability of the MiSeq-HyDRA platform to detect minority variants present as low as 0.5% in mixed plasmid populations, which is consistent with other studies reporting on MiSeq sensitivity^39^.

Accurate detection of LADRVs using NGS is largely dependent on the amount of original template used in the assay. The actual RNA copy number in the PCR is determined by the VL, specimen storage condition, extraction method, and accurate laboratory technique. We have shown the influence of VL on the detection of LADRVs using serial dilutions of specimens with known VL. The ability of the MiSeq-HyDRA platform to correctly identify LADRVs and distinguish between false-positive and false-negative results is directly correlated to the VL of the specimen. In addition, previous studies have shown that in plasma samples with less than 10,000 HIV RNA cp/ml, PCR bias can occur causing the output of ultra-deep sequencing to be reflective of only a small proportion of PCR amplicons, skewing allelic frequencies, and resulting in a misrepresentation of the true variant alleles in the HIV quasispecies^13,45^.

The “one specimen, one sequence” model with SS has long been utilized as the gold standard for HIVDR genotyping and many downstream data mining strategies have been developed based on this paradigm. HyDRA Web fulfills the “one sequence, one specimen” model by allowing for Sanger-like consensus sequences to be generated from the MiSeq sequencing data at a user-defined threshold. Consensus sequences derived at three different mixed-base thresholds (20%, 15%, 10%) from the MiSeq-HyDRA assay have >99% concordance with corresponding Sanger sequences, as well as high intra-assay precision and inter-assay reproducibility. Determining the clinical relevance of minor variants, LADRVs in particular, and the ideal cut-off for reporting HIVDR is critical to defining a threshold for MiSeq-derived consensus sequences.

In addition to producing valid data, the reagent cost and turnaround time to run a test are important when considering its feasibility for use in routine HIVDR genotyping. As compared to SS assay, the MiSeq-HyDRA platform may lead to an approximately 10% reduction of the reagent cost and labour intensity or turnaround time when a batch of 96 specimens are being processed (Supplementary Table S6). The per sample cost could be further reduced by 30% if amplicon sequencing approach is applied, which bypasses the usage of the expensive Nextera XT library kit^46^. Notably, such cost and labour savings could be achieved only when specimens are processed in batches using MiSeq-HyDRA protocol. Hence, rather than individualized clinical testing, Miseq-HyDRA platform is more suited to HIVDR surveillance testing which usually involves large sizes of samples being processed.

In summary, we demonstrate a successful and valid HIV drug resistance genotyping protocol for the Illumina MiSeq to be used in conjunction with our custom data analysis pipeline, HyDRA Web. The protocol we have described here accurately and reproducibly identifies HIVDR mutations comparable with SS results, encompassing a broad subtype coverage to a sensitivity of 500 cp/ml. We have demonstrated that the MiSeq-HyDRA platform performs with a low 0.21% error rate in both homopolymer and SDRM regions and it is capable of detecting minority variants down to 0.5% in a mixed population. Consensus sequences derived from the MiSeq-HyDRA assay have > 99% concordance with corresponding Sanger sequences, as well as high intra-assay precision and inter-assay reproducibility. Using our MiSeq protocol in conjunction with HyDRA Web results in high quality data output which can be used in labs worldwide to produce complete and individualized HIV-1 surveillance drug resistance reports.

## Supplementary information

Supplement information

## Data Availability

All data generated or analyzed during this study are included in this published article and its Supplementary Information Files or would be available upon reasonable request.
