# Supplementary material for: A MiSeq-HyDRA platform for enhanced HIV drug resistance genotyping and surveillance
Source: Sci Rep. 2019 Jun 20;9:8970. doi: 10.1038/s41598-019-45328-3 (PMC6586679; doi:10.1038/s41598-019-45328-3)
Supplement: Supplementary file 1 — Supplement information [file 41598_2019_45328_MOESM1_ESM.docx]

**Supplementary Information**

**A MiSeq-HyDRA platform for enhanced HIV drug resistance genotyping and surveillance**

Tracy Taylor^1^, Emma R. Lee^1^, Mikaela Nykoluk^1^, Eric Enns^2^, Ben Liang^2^, Rupert Capina^1^, Marie-Krystel Gauthier^1^, Gary Van Domselaar^2,3^, Paul Sandstrom^1,3^, James Brooks^1^, Hezhao Ji^1,3*^

^1^National HIV and Retrovirology Laboratories, National Microbiology Laboratory at JC Wilt Infectious Diseases Research Centre, Public Health Agency of Canada, Winnipeg, Canada;

^2^Bioinformatics Core, National Microbiology Laboratory, Public Health Agency of Canada, Winnipeg, Canada;

^3^Department of Medical Microbiology and Infectious Diseases, University of Manitoba, Winnipeg, Canada;

***Corresponding author at:**

National HIV & Retrovirology Laboratories,

Public Health Agency of Canada,

1015 Arlington Street

Winnipeg, MB, Canada, R3E 3R2

Tel: 1-204-789-6521; Fax: 1-204-318-2221

E-mail: hezhao.ji@canada.ca

**SUPPLEMENTAL DATA**

| **Supplementary Table S1.** Plasmid error rates for the MiSeq-HyDRA platform**.** | | | | | | | |
| --- | --- | --- | --- | --- | --- | --- | --- |
| Plasmid | **Intra-assay Precision (run 1)** | | | **Inter-assay Reproducibility** | | | MiSeq Run |
|  | Full Amplicon (974 nt) | SDRM sites (n=43) | Homo-polymeric sites (n=20) | Full Amplicon (974 nt) | SDRM sites (n=43) | Homo-polymeric sites (n=20) |  |
| P1 | 0.0020 | 0.0021 | 0.0024 | 0.0021 | 0.0021 | 0.0024 | run 2 |
|  | 0.0020 | 0.0020 | 0.0023 | 0.0021 | 0.0023 | 0.0023 | run 3 |
|  | 0.0022 | 0.0022 | 0.0026 | 0.0025 | 0.0027 | 0.0020 | run 4 |
| P2 | 0.0022 | 0.0022 | 0.0025 | 0.0021 | 0.0021 | 0.0024 | run 2 |
|  | 0.0020 | 0.0020 | 0.0023 | 0.0019 | 0.0020 | 0.0021 | run 3 |
|  | 0.0022 | 0.0022 | 0.0025 | 0.0014 | 0.0013 | 0.0013 | run 4 |
| **Mean** | **0.0021** | **0.0021** | **0.0024** | **0.0020** | **0.0021** | **0.0021** |  |
| **StDev** | **0.0001** | **0.0001** | **0.0001** | **0.0003** | **0.0005** | **0.0004** |  |
| **IQR %** | **0.13** | **0.13** | **0.14** | **0.13** | **0.14** | **0.13** |  |
| **CV %** | **4.86** | **4.98** | **4.38** | **17.07** | **22.30** | **20.18** |  |

| **Supplementary Table S2.** Comparison of sequencing methodologies using percent nucleotide identity between MiSeq-HyDRA MBIT consensus sequences and corresponding Sanger-derived consensus sequence. | | | |
| --- | --- | --- | --- |
|  | MiSeq-Sanger Nucleotide Identity (%) | | |
| Sample | ≥ 20% | ≥ 15% | ≥ 10% |
| SDR-6 | 99.90 | 99.59 | 99.79 |
| SDR-8 | 99.07 | 100.00 | 100.00 |
| SDR-12 | 99.90 | 99.59 | 99.49 |
| SDR-16 | 100.00 | 99.28 | 99.79 |
| SDR-18 | 99.90 | 99.90 | 97.22 |
| SDR-23 | 100.00 | 99.18 | 98.66 |
| SDR-30 | 100.00 | 99.38 | 99.79 |
| SDR-31 | 100.00 | 99.90 | 99.07 |
| SDR-32 | 99.28 | 99.90 | 100.00 |
| SDR-33 | 100.00 | 99.90 | 98.97 |
| SDR-34 | 99.18 | 99.49 | 98.25 |
| SDR-36 | 100.00 | 99.59 | 99.59 |
| SDR-37 | 99.49 | 99.69 | 97.74 |
| SDR-48 | 99.69 | 98.56 | 99.79 |
| SDR-55 | 100.00 | 99.90 | 98.77 |
| SDR-63 | 99.59 | 100.00 | 100.00 |
| SDR-79 | 99.28 | 100.00 | 99.18 |
| SDR-81 | 98.97 | 99.07 | 99.38 |
| SDR-89 | 100.00 | 98.46 | 99.28 |
| SDR-93 | 99.07 | 99.49 | 99.69 |
| SDR-94 | 99.90 | 100.00 | 99.90 |
| SDR-95 | 99.90 | 99.49 | 99.49 |
| SDR-96 | 98.97 | 99.18 | 99.28 |
| SDR-98 | 99.59 | 99.79 | 99.90 |
| SDR-101 | 99.90 | 100.00 | 100.00 |
| SDR-102 | 98.97 | 99.49 | 99.69 |
| SDR-110 | 100.00 | 100.00 | 99.28 |
| SDR-127 | 98.46 | 99.49 | 100.00 |
| SDR-140 | 99.49 | 100.00 | 99.38 |
| SDR-143 | 99.69 | 99.38 | 98.56 |
| SDR-150 | 100.00 | 100.00 | 99.38 |
| SDR-162 | 99.59 | 98.97 | 100.00 |
| **Average** | **99.62** | **99.58** | **99.35** |
| Total # mismatches including mixed bases | 146 | 157 | 214 |
| Total # mismatches due to complete base change | 13 | 10 | 9 |

| **Supplementary Table S3.** Summary of HIVDR mutation frequencies (%) identified by the MiSeq-HyDRA platform, compared to those detected (YES) or not detected (NO) by the Sanger sequencing (SS) method. (*surveillance DRMs are in* ***bold***) | | | | | | |
| --- | --- | --- | --- | --- | --- | --- |
| Sample ID | Clade | Gene | DRM | Classification | MiSeq Frequency (%) | Detected by SS |
| SDR-110 | C | RT | **Y188L** | NNRTI | 99.67 | YES |
| SDR-63 | A | RT | V179I | Other | 99.65 | YES |
| SDR-110 | C | RT | V106I | NNRTI | 99.56 | YES |
| SDR-37 | B | RT | **K101P** | NNRTI | 99.53 | YES |
| SDR-31 | CRF02_AG | PR | L10I | Other | 99.53 | YES |
| SDR-31 | CRF02_AG | PR | K20I | Other | 99.53 | YES |
| SDR-150 | B | RT | V179I | Other | 99.52 | YES |
| SDR-16 | CRF06_cpx | PR | L10I | Other | 99.52 | YES |
| SDR-8 | B | PR | A71V | Other | 99.52 | YES |
| SDR-48 | B | RT | **G190A** | NNRTI | 99.51 | YES |
| SDR-140 | B | PR | L10I | Other | 99.47 | YES |
| VQA29-5 | B | PR | **V82A** | Major | 99.44 | YES |
| SDR-32 | D | PR | L10I | Other | 99.41 | YES |
| SDR-6 | B | RT | **K103N** | NNRTI | 99.40 | YES |
| SDR-3 | G | PR | K20I | Other | 99.36 | YES |
| SDR-6 | B | PR | A71T | Other | 99.36 | YES |
| SDR-115 | C | PR | L10I | Other | 99.34 | YES |
| SDR-55 | G/C | RT | **M184V** | NRTI | 99.34 | YES |
| SDR-112 | A1/D | RT | **Y181C** | NNRTI | 99.26 | YES |
| SDR-63 | A | RT | K101R | Other | 99.26 | YES |
| SDR-55 | G/B | PR | V82I | Other | 99.25 | YES |
| VQA29-4 | B | PR | **L23I** | Accessory | 99.23 | YES |
| SDR-95 | CRF02_AG | PR | K20I | Other | 99.18 | YES |
| VQA29-4 | B | PR | **I54M** | Major | 99.17 | YES |
| SDR-89 | A | RT | V179I | Other | 99.16 | YES |
| SDR-113 | B | PR | L10V | Other | 99.12 | YES |
| SDR-143 | C | RT | **K103N** | NNRTI | 99.11 | YES |
| SDR-119 | B | PR | L33I | Other | 99.11 | YES |
| VQA29-3 | C | RT | **M184V** | NRTI | 99.09 | YES |
| SDR-18 | C | PR | T74S | Pther | 99.05 | YES |
| VQA29-4 | B | PR | K20R | Other | 99.05 | YES |
| SDR-150 | B | RT | **K103N** | NNRTI | 99.04 | YES |
| VQA29-5 | B | PR | **M46I** | Major | 99.03 | YES |
| VQA30-3 | B | RT | **T215Y** | NRTI | 99.03 | YES |
| VQA29-3 | C | PR | T74S | Other | 99.03 | YES |
| SDR-79 | B | RT | **T215S** | NRTI | 99.02 | YES |
| SDR-150 | B | PR | A71T | Other | 99.02 | YES |
| SDR-140 | B | RT | **K103N** | NNRTI | 99.01 | YES |
| SDR-099 | C | PR | K20R | Other | 98.97 | YES |
| SDR-110-13 | B | PR | L10I | Other | 98.97 | YES |
| SDR-95 | CRF02_AG | PR | V11I | Other | 98.97 | YES |
| SDR-55 | G/B | PR | K20I | Other | 98.97 | YES |
| SDR-102 | B | RT | V106I | NNRTI | 98.95 | YES |
| SDR-3 | G | PR | V82I | Other | 98.95 | YES |
| SDR-101 | B | RT | **K103S** | NNRTI | 98.94 | YES |
| VQA30-1 | A | PR | K20R | Other | 98.94 | YES |
| VQA29-5 | B | RT | **M41L** | NRTI | 98.92 | YES |
| SDR-094 | B | RT | V106I | NNRTI | 98.90 | YES |
| SDR-105 | B | PR | V82I | Other | 98.90 | YES |
| VQA29-4 | B | PR | **L90M** | Major | 98.87 | YES |
| VQA30-3 | B | PR | **V32I** | Major | 98.87 | YES |
| SDR-107 | B | RT | **K103N** | NNRTI | 98.86 | YES |
| SDR-117 | B | RT | **V75M** | NRTI | 98.86 | YES |
| VQA29-4 | B | PR | L33F | Accessory | 98.85 | YES |
| VQA29-5 | B | PR | **V32I** | Major | 98.84 | YES |
| VQA29-4 | B | PR | **F53L** | Major | 98.82 | YES |
| VQA29-4 | B | RT | A98G | NNRTI | 98.81 | YES |
| VQA30-3 | B | PR | **I84V** | Major | 98.80 | YES |
| SDR-097 | A1/D | PR | L10V | Other | 98.78 | YES |
| SDR-100 | A1 | PR | L10I | Other | 98.77 | YES |
| SDR-112 | A1/D | RT | **G190A** | NNRTI | 98.76 | YES |
| SDR-117 | B | PR | L33I | Other | 98.76 | YES |
| VQA29-5 | B | PR | K43T | Accessory | 98.76 | YES |
| VQA30-5 | C | RT | E138K | NNRTI | 98.76 | YES |
| SDR-63 | A | PR | L10V | Other | 98.75 | YES |
| VQA29-5 | B | PR | L33F | Accessory | 98.75 | YES |
| SDR-117 | B | PR | Q58E | Accessory | 98.74 | YES |
| VQA29-4 | B | PR | **M46I** | Major | 98.72 | YES |
| SDR-121 | A1 | PR | K20R | Other | 98.72 | YES |
| VQA30-3 | B | PR | A71V | Other | 98.72 | YES |
| VQA29-4 | B | RT | V179I | Other | 98.71 | YES |
| VQA30-3 | B | PR | **M46I** | Major | 98.69 | YES |
| VQA30-3 | B | PR | **V82A** | Major | 98.68 | YES |
| SDR-121 | A1 | RT | V179I | Other | 98.68 | YES |
| VQA30-3 | B | RT | **M184V** | NRTI | 98.67 | YES |
| SDR-33 | B | RT | **L210W** | NRTI | 98.66 | YES |
| SDR-106 | B | RT | V106I | Other | 98.66 | YES |
| VQA29-4 | B | RT | **Y181C** | NNRTI | 98.63 | YES |
| VQA29-4 | B | PR | **V32I** | Major | 98.63 | YES |
| SDR-6 | B | RT | V179I | Other | 98.62 | YES |
| SDR-79 | B | RT | **M41L** | NRTI | 98.61 | YES |
| VQA29-4 | B | RT | **K219E** | NRTI | 98.61 | YES |
| SDR-33 | B | RT | **M41L** | NRTI | 98.59 | YES |
| VQA29-4 | B | RT | **M184V** | NRTI | 98.58 | YES |
| VQA29-5 | B | RT | **Y188L** | NNRTI | 98.58 | YES |
| VQA29-4 | B | RT | T69G | NRTI | 98.58 | YES |
| VQA29-5 | B | RT | **M184V** | NRTI | 98.57 | YES |
| VQA29-4 | B | RT | A71V | Other | 98.55 | YES |
| SDR-33 | B | RT | **T215S** | NRTI | 98.54 | YES |
| SDR-117 | B | RT | T69S | Other | 98.51 | YES |
| VQA29-5 | B | RT | **T215Y** | NRTI | 98.50 | YES |
| SDR-37 | B | RT | V179I | Other | 98.46 | YES |
| SDR-113 | B | PR | A71T | Other | 98.40 | YES |
| VQA29-5 | B | RT | V179I | Other | 98.39 | YES |
| VQA29-4 | B | RT | **K70R** | NRTI | 98.38 | YES |
| SDR-100 | A1 | RT | V179I | Other | 98.33 | YES |
| SDR-140 | B | RT | **P225H** | NNRTI | 98.29 | YES |
| VQA29-4 | B | RT | V108I | NNRTI | 98.29 | YES |
| VQA29-4 | B | PR | G73S | Accessory | 98.25 | YES |
| VQA29-5 | B | RT | **L210W** | NRTI | 98.25 | YES |
| VQA29-5 | B | PR | A71V | Other | 98.25 | YES |
| SDR-109 | CRF01_AE | RT | V106I | Other | 98.24 | YES |
| VQA30-3 | B | RT | **L210W** | NRTI | 98.19 | YES |
| VQA29-4 | B | PR | K43T | Accessory | 98.18 | YES |
| VQA29-5 | B | PR | **I84V** | Major | 98.12 | YES |
| VQA30-3 | B | RT | **Y188L** | NNRTI | 98.11 | YES |
| VQA30-2 | C | RT | **K103N** | NNRTI | 98.06 | YES |
| SDR-102 | B | PR | L10V | Other | 98.03 | YES |
| SDR-81 | C | RT | V179D | NNRTI | 98.02 | YES |
| VQA29-4 | B | PR | **I47V** | Major | 98.01 | YES |
| SDR-117 | B | PR | A71V | Other | 98.00 | YES |
| VQA29-4 | B | RT | V90I | Other | 97.97 | YES |
| VQA30-3 | B | PR | K43T | Other | 97.97 | YES |
| SDR-79 | B | RT | **L210W** | NRTI | 97.93 | YES |
| SDR-112 | A1/D | RT | **K101E** | NNRTI | 97.87 | YES |
| SDR-093 | B | PR | L10I | Other | 97.84 | YES |
| SDR-37 | B | PR | L10I | Other | 97.81 | YES |
| SDR-122 | B | PR | V82I | Other | 97.69 | YES |
| VQA30-1 | A | RT | V179I | Other | 97.69 | YES |
| SDR-109 | CRF01_AE | RT | K238R | Other | 97.54 | YES |
| SDR-111 | C | PR | L10I | Other | 97.28 | YES |
| VQA30-3 | B | PR | L33F | Accessory | 97.27 | YES |
| VQA30-5 | C | RT | **K101E** | NNRTI | 97.02 | YES |
| VQA30-3 | B | RT | V179I | Other | 96.34 | YES |
| SDR-096 | B | PR | L10I | Other | 96.30 | YES |
| SDR-16 | CRF06_cpx | PR | K20I | Other | 96.30 | YES |
| VQA30-3 | B | RT | **M41L** | NRTI | 96.24 | YES |
| SDR-103 | B | PR | A71V | Other | 96.12 | YES |
| SDR-162 | B | RT | V106I | Other | 95.87 | YES |
| VQA29-4 | B | RT | **T215V** | NRTI | 94.01 | YES |
| SDR-127 | C | PR | K20R | Other | 92.07 | YES |
| SDR-37 | B | RT | K103R | Other | 90.03 | YES |
| VQA29-3 | C | RT | **D67N** | NRTI | 88.52 | YES |
| SDR-37 | B | PR | A71V | Other | 88.31 | YES |
| VQA29-3 | C | RT | **K70R** | NRTI | 83.21 | YES |
| VQA30-3 | B | PR | L10V | Other | 82.39 | YES |
| SDR-81 | C | PR | V82I | Other | 77.11 | YES |
| SDR-102 | B | RT | K101R | Other | 75.53 | YES |
| VQA29-1 | B | RT | **K103N** | NNRTI | 70.20 | YES |
| VQA29-5 | B | PR | **I54V** | Major | 70.08 | YES |
| VQA30-3 | B | PR | **I54V** | Major | 67.80 | YES |
| VQA29-5 | B | PR | L10V | Other | 62.20 | YES |
| SDR-109 | CRF01_AE | PR | L10V | Other | 62.05 | YES |
| VQA30-4 | B | PR | L10I | Other | 55.24 | YES |
| SDR-110-13 | B | RT | K101R | Other | 52.51 | YES |
| VQA29-4 | B | RT | **G190A** | NNRTI | 49.70 | YES |
| VQA29-2 | B | RT | **M184V** | NRTI | 48.08 | YES |
| VQA29-1 | B | RT | L210F | Other | 47.01 | YES |
| SDR-95 | CRF02_AG | PR | V82I | Other | 43.05 | YES |
| VQA29-1 | B | RT | V90I | Other | 38.82 | YES |
| VQA29-4 | B | PR | L10F | Accessory | 34.99 | YES |
| SDR-112 | A1/D | **RT** | **L74I** | NRTI | 34.70 | YES |
| VQA29-3 | C | RT | V90I | Other | 33.18 | YES |
| VQA30-3 | B | PR | **I54L** | Major | 30.68 | YES |
| VQA29-5 | B | PR | **I54L** | Major | 29.21 | YES |
| VQA29-4 | B | PR | L10V | Other | 27.66 | YES |
| SDR-95 | CRF02_AG | RT | K101R | Other | 24.93 | YES |
| SDR-95 | CRF02_AG | PR | L33F | Accessory | 24.41 | YES |
| SDR-102 | B | RT | **G190A** | NNRTI | 21.02 | YES |
| SDR-121 | A1 | RT | V106I | Other | 19.85 | YES |
| SDR-36 | B | RT | **K219Q** | NRTI | 18.93 | YES |
| SDR-95 | CRF02_AG | PR | L33F | Accessory | 17.54 | YES |
| SDR-112 | A1/D | RT | T69N | Other | 17.22 | YES |
| VQA30-3 | B | PR | L10I | Other | 15.91 | YES |
| SDR-95 | CRF02_AG | RT | K101R | Other | 10.68 | YES |
| SDR-117 | B | RT | **L210W** | NRTI | 9.21 | YES |
| VQA29-5 | B | PR | L10I | Other | 37.15 | NO |
| SDR-112 | A1/D | RT | E138K | NNRTI | 29.41 | NO |
| SDR-89 | A | PR | K20R | Other | 26.11 | NO |
| SDR-109 | CRF01_AE | PR | L10I | Other | 17.30 | NO |
| SDR-119 | B | PR | **M46I** | Major | 15.57 | NO |
| SDR-34 | F | RT | V106I | Other | 15.08 | NO |
| SDR-102 | B | RT | V179I | Other | 13.30 | NO |
| SDR-6 | B | PR | V11I | Other | 10.23 | NO |
| VQA29-3 | C | RT | **M41L** | NRTI | 8.27 | NO |
| VQA29-5 | B | PR | L24M | Accessory | 8.13 | NO |
| VQA29-4 | B | RT | **K101E** | NNRTI | 8.12 | NO |
| SDR-30 | C | PR | K20R | Other | 8.05 | NO |
| SDR-121 | A1 | RT | E138K | NNRTI | 6.98 | NO |
| SDR-093 | B | RT | V106I | Other | 6.26 | NO |
| SDR-63 | A | RT | **K219E** | NRTI | 6.23 | NO |
| SDR-37 | B | RT | E138K | NNRTI | 6.13 | NO |
| SDR-48 | B | RT | V108I | NNRTI | 4.78 | NO |
| SDR-32 | D | RT | K103R | Other | 4.46 | NO |
| VQA29-4 | B | RT | **T215I** | NRTI | 4.27 | NO |
| VQA30-3 | B | PR | L24M | Accessory | 4.24 | NO |
| SDR-6 | B | RT | **K103S** | NNRTI | 4.06 | NO |
| SDR-121 | A1 | RT | **L100I** | NNRTI | 3.90 | NO |
| VQA29-3 | C | RT | **T215F** | NRTI | 3.69 | NO |
| SDR-110-13 | B | RT | V90I | Other | 3.62 | NO |
| SDR-096 | B | PR | V82I | Other | 3.59 | NO |
| VQA29-3 | C | RT | **K219Q** | NRTI | 3.54 | NO |
| SDR-16 | CRF06_cpx | PR | K20V | Other | 3.38 | NO |
| VQA30-1 | A | PR | **M46I** | Major | 3.23 | NO |
| SDR-101 | B | RT | E138K | NNRTI | 3.22 | NO |
| SDR-113 | B | RT | **L210W** | NRTI | 3.14 | NO |
| SDR-3 | G | RT | V118I | Other | 3.07 | NO |
| SDR-122 | B | PR | **M46I** | Major | 2.94 | NO |
| SDR-16 | CRF06_cpx | RT | K238R | Other | 2.90 | NO |
| SDR-55 | G/B | RT | V90I | NNRTI | 2.83 | NO |
| VQA30-1 | A | RT | **M184I** | NRTI | 2.60 | NO |
| SDR-55 | G/B | RT | L210S | Other | 2.44 | NO |
| SDR-107 | B | PR | V11I | Other | 2.44 | NO |
| SDR-3 | G | PR | **I54T** | Major | 2.40 | NO |
| SDR-127 | C | RT | T69N | Other | 2.38 | NO |
| VQA29-5 | B | RT | **K103N** | NNRTI | 2.35 | NO |
| SDR-096 | B | PR | A71V | Other | 1.95 | NO |
| SDR-121 | A1 | RT | **G190E** | NNRTI | 1.90 | NO |
| SDR-34 | F | RT | V90I | NNRTI | 1.83 | NO |
| SDR-36 | B | PR | L33I | Other | 1.78 | NO |
| SDR-113 | B | PR | V11I | Other | 1.77 | NO |
| VQA29-2 | B | RT | E138K | NNRTI | 1.69 | NO |
| SDR-102 | B | RT | V75I | NRTI | 1.64 | NO |
| SDR-107 | B | RT | K238N | NNRTI | 1.61 | NO |
| SDR-111 | C | RT | **G190E** | NNRTI | 1.58 | NO |
| VQA30-5 | C | RT | **P225H** | NNRTI | 1.54 | NO |
| VQA30-4 | B | RT | **L74V** | NRTI | 1.52 | NO |
| SDR-119 | B | RT | K70S | NRTI | 1.51 | NO |
| VQA29-1 | B | RT | V179I | Other | 1.50 | NO |
| SDR-107 | B | PR | **I84V** | Major | 1.46 | NO |
| SDR-79 | B | RT | T69I | Other | 1.46 | NO |
| SDR-107 | B | PR | **I50V** | Major | 1.45 | NO |
| VQA30-4 | B | PR | **I85V** | Other | 1.44 | NO |
| SDR-096 | B | PR | K20R | Other | 1.44 | NO |
| SDR-23 | C | RT | **K65R** | NRTI | 1.40 | NO |
| VQA29-1 | B | RT | T69S | Other | 1.39 | NO |
| VQA30-4 | B | RT | **D67E** | NRTI | 1.33 | NO |
| VQA30-4 | B | PR | L33I | Other | 1.33 | NO |
| SDR-110-13 | B | PR | V11I | Other | 1.32 | NO |
| SDR-102 | B | RT | L210F | Other | 1.28 | NO |
| VQA30-1 | A | PR | L33V | Other | 1.26 | NO |
| VQA30-3 | B | RT | **K103N** | NNRTI | 1.23 | NO |
| VQA30-5 | C | RT | **D67E** | NRTI | 1.23 | NO |
| SDR-096 | B | PR | **M46L** | Major | 1.21 | NO |
| SDR-119 | B | PR | **D30N** | Major | 1.19 | NO |
| VQA30-2 | C | PR | L10R | Other | 1.19 | NO |
| SDR-119 | B | RT | M41I | Other | 1.18 | NO |
| SDR-48 | B | RT | K238R | Other | 1.18 | NO |
| SDR-110-13 | B | RT | M41I | Other | 1.16 | NO |
| SDR-89 | A | RT | **D67E** | NRTI | 1.13 | NO |
| VQA30-1 | A | RT | **P225H** | NNRTI | 1.12 | NO |
| VQA30-5 | C | RT | **L74V** | NRTI | 1.12 | NO |
| SDR-31 | CRF02_AG | RT | M41I | Other | 1.11 | NO |
| SDR-63 | A | RT | **D67E** | NRTI | 1.07 | NO |
| SDR-63 | A | RT | **M184V** | NRTI | 1.07 | NO |
| SDR-098 | B | RT | K238R | Other | 1.07 | NO |
| SDR-36 | B | RT | T69N | Other | 1.04 | NO |
| VQA30-5 | C | PR | L10R | Other | 1.03 | NO |
| SDR-79 | B | RT | G190V | NNRTI | 1.02 | NO |
| SDR-16 | CRF06_cpx | RT | V106I | Other | 1.01 | NO |

| **Supplementary Table S4.** Intra-assay precision and inter-assay reproducibility of the MiSeq-HyDRA platform as measured by the percent identity of nucleotide and amino acid consensus sequences at three different mixed base identity thresholds. | | | | | | |
| --- | --- | --- | --- | --- | --- | --- |
| Average Percent Identity | INTRA-ASSAY (n=7 triplicate samples) | | | INTER-ASSAY (n=7 in triplicate runs) | | |
|  | **≥ 20%** | **≥ 15%** | **≥ 10%** | **≥ 20%** | **≥ 15%** | **≥ 10%** |
| Nucleotide | 99.82 | 99.72 | 99.46 | 99.54 | 99.40 | 98.97 |
| Amino Acid | 99.98 | 99.91 | 99.87 | 99.86 | 99.89 | 99.83 |

| **Supplementary Table S5.** Inter-assay reproducibility for LADRVs identified in 3 independent MiSeq-HyDRA assays. | | | | | | | | | |
| --- | --- | --- | --- | --- | --- | --- | --- | --- | --- |
|  | | | | | | Frequency (%) | | |  |
| Sample Name | Gene | Class | Wild Type | Position | Mutation | Run 1 | Run 2 | Run 3 | SS* |
| SDR-6 | PR | Accessory | V | 11 | I | ND | **10.23** | ND | No |
|  | PR | **Major** | M | 46 | L | ND | ND | **5.07** | No |
|  | PR | Accessory | A | 71 | T | 99.36 | 99.36 | 98.98 | Yes |
|  | RT | **NRTI** | L | 74 | V | ND | ND | **1.27** | No |
|  | RT | **NNRTI** | K | 103 | N | 99.4 | 95.45 | 99.04 | Yes |
|  | RT | **NNRTI** | K | 103 | S | **4.06** | ND | ND | No |
|  | RT | Other | V | 179 | I | 98.62 | 98.67 | 96.52 | Yes |
| SDR-16 | PR | Accessory | L | 10 | I | 99.52 | 99.61 | 99.39 | Yes |
|  | PR | Other | K | 20 | I | 96.3 | 95.59 | 95.73 | Yes |
|  | PR | Other | K | 20 | V | **3.38** | **4.03** | **3.59** | No |
|  | PR | **Major** | M | 46 | I | **1.16** | ND | ND | No |
|  | PR | Other | V | 82 | I | ND | ND | **2.43** | No |
|  | RT | NNRTI | V | 106 | I | **1.01** | **1.36** | **1.97** | No |
|  | RT | Other | K | 238 | R | **2.9** | ND | ND | No |
| SDR-32 | PR | Accessory | L | 10 | I | 99.41 | 99.14 | 99.02 | Yes |
|  | RT | Other | K | 101 | R | ND | **1.51** | ND | No |
|  | RT | Other | K | 103 | R | **4.46** | ND | **3.47** | No |
| SDR-36 | PR | Accessory | V | 11 | I | ND | 1.14 | **1.21** | No |
|  | PR | Other | L | 33 | I | **1.78** | **1.47** | **1.05** | No |
|  | PR | Accessory | A | 71 | T | 99.33 | 99.47 | 98.74 | Yes |
|  | RT | NRTI | T | 69 | N | **1.04** | **1.32** | **1.43** | No |
|  | RT | **NRTI** | K | 219 | Q | **18.93** | **19.08** | **21.2** | Yes |
| SDR-37 | PR | Accessory | L | 10 | I | 97.81 | 98.58 | 98.95 | Yes |
|  | PR | Accessory | A | 71 | V | 88.31 | 88.82 | 88.25 | Yes |
|  | RT | **NRTI** | L | 74 | V | ND | ND | **1.12** | No |
|  | RT | **NNRTI** | K | 101 | P | 99.53 | 99.52 | 99.25 | Yes |
|  | RT | Other | K | 103 | R | 90.03 | 91.62 | 92.5 | Yes |
|  | RT | NNRTI | E | 138 | K | **6.13** | **5.9** | **5.6** | No |
|  | RT | Other | V | 179 | I | 98.46 | 98.5 | 97.03 | Yes |
| SDR-89 | PR | Accessory | K | 20 | R | **26.11** | **19.86** | **20.71** | No |
|  | RT | **NRTI** | D | 67 | E | 1.13 | ND | ND | No |
|  | RT | Other | V | 179 | I | 99.16 | 98.03 | 97.64 | Yes |
| SDR-95 | PR | Accessory | V | 11 | I | 98.97 | 96.59 | 96.98 | Yes |
|  | PR | Other | K | 20 | I | 99.18 | 97.07 | 96.94 | Yes |
|  | PR | Accessory | K | 20 | T | ND | **2.2** | **2.32** | No |
|  | PR | Other | L | 33 | F | **24.41** | **17.54** | **18.15** | Yes |
|  | PR | **Major** | M | 46 | I | ND | **1.47** | ND | No |
|  | PR | Other | V | 82 | I | 43.05 | 44.48 | 43.74 | Yes |
|  | PR | Other | L | 89 | I | ND | **2.91** | **2.75** | No |
|  | RT | Other | K | 101 | R | **24.93** | **10.68** | **10.69** | Yes |
|  | RT | NNRTI | V | 106 | I | ND | **1.55** | ND | No |
|  | RT | NNRTI | V | 108 | I | ND | **1.04** | ND | No |

| **Supplementary Table S6.** Cost and labour comparison between SS, and the MiSeq protocol. *Cost analysis begins after the shared steps of RNA extraction and RT-PCR and does not include the initial start-up costs of purchasing the machines.* | | |
| --- | --- | --- |
|  | **SS** | **MiSeq Nextera** |
|  | Cost (CAD$/sample) | Cost (CAD$/sample) |
| Nested PCR | $3.11 | $2.19 |
| Qiaxcel | $0.64 | $0.64 |
| PCR Clean Up | $1.54 | n/a |
| Sanger Sequencing | $71.63 | n/a |
| Index PCR | n/a | $3.06 |
| AMPure purification | n/a | $1.00 |
| Nextera XT Kit | n/a | $44.20 |
| Qubit | n/a | $0.60 |
| MiSeq v3 kit | n/a | n/a |
| MiSeq v2 kit | n/a | $16.72 |
| **Total** | **76.92** | **68.41** |
|  | Labor (hours/96 samples) | Labor (hours/96 samples) |
| Hands-on Time | 35 | 26 |
| Processing Time | 32 | 40 |
| Analysis Time | 16 | 8 |
| **Total** | **83** | **74** |
